# College Students Recognize Characteristics of Autism, but Struggle to Differentiate Between Characteristics of Autism and Other Disabilities

**DOI:** 10.1007/s10803-024-06631-9

**Published:** 2024-11-15

**Authors:** Camilla M. McMahon

**Affiliations:** Department of Social and Behavioral Sciences, Miami University, 1601 University Blvd., Hamilton, OH 45011 USA

**Keywords:** Autism knowledge, Autistic characteristics or symptomatology, Disability characteristics or symptomatology, Metacognition, Confidence, Perceived knowledge

## Abstract

The current study evaluates whether college students can identify characteristics of autism as diagnostic for autism, and characteristics of other disabilities as not diagnostic for autism. This study also examines metacognitive awareness of autism knowledge, evaluating whether college students can accurately calibrate their confidence in their beliefs about autistic characteristics. 283 college students completed the Autism Symptomatology Knowledge Assessment (McMahon et al. Research in Autism Spectrum Disorders 71:101499, 2020). In this assessment, participants were presented with characteristics of autism and other disabilities and asked to identify which characteristics could be used to diagnose someone with autism. For each characteristic, participants indicated how certain they were in their response. Participants more accurately categorized characteristics of autism, particularly social interaction and communication challenges, as being consistent with an autism diagnosis. Participants had more difficulty identifying that characteristics of other disabilities, especially anxiety, ADHD, and learning disabilities, were not diagnostic for autism. For autistic characteristics, participants’ confidence and accuracy were positively correlated, such that participants who responded accurately were more confident in their response than those who responded inaccurately. For other disability characteristics, confidence and accuracy were typically not correlated or inversely correlated, indicating poor metacognitive awareness. College students confuse autism with other disabilities, which may have important implications in real-world contexts. Furthermore, individuals with poor metacognitive awareness of their autism knowledge may not realize that they are confusing autism with other disabilities, such that they may not seek out additional corrective information about autism.

With the advent of social media, it has become much easier to access health-related information, including information about mental health and disabilities. However, the information shared on social media is not always accurate and includes content that is blatantly misleading and false, as well as content that is ambiguous or partially true (Starvaggi et al., 2024). For instance, Yeung et al. (2022) examined the quality of the 100 most popular TikTok videos about ADHD and classified 52% of the videos as misleading and only 21% of the videos as useful. Similarly, Aragon-Guevara et al. (2023) reviewed 133 informational videos about autism on TikTok and categorized 41% of the videos as inaccurate, 32% of the videos as overgeneralizing the experience of some autistic individuals^1^ to all autistic individuals, and only 27% of the videos as accurate.

While misinformation about autism is widespread, it is also becoming increasingly clear that accurate knowledge about autism is critical for reducing stigma and improving the quality of interactions between autistic and non-autistic individuals. For example, Sasson and Morrison (2019) found that participants who were more knowledgeable about autism had more favorable first impressions of autistic individuals featured on a short video clip and labeled as being autistic. McMahon et al. (2021) asked participants to read a series of vignettes featuring individuals with and without autistic characteristics interviewing for jobs, and participants who were more knowledgeable about autism were more likely to recommend the candidate for the job, perceive positive personality traits in the candidate, and anticipate a high-quality work performance from the candidate, particularly when an autism diagnosis was disclosed. Similarly, Romualdez et al. (2021) conducted interviews with adults with autism about their employment experiences, and the interview themes showed that disclosing an autism diagnosis at work was a more positive experience when employers and colleagues had prior knowledge and understanding of autism.

Given the importance of autism knowledge in promoting positive interactions between autistic and non-autistic individuals, several studies have also begun to examine the effect of autism knowledge interventions. Autism knowledge interventions have been shown to increase participants’ knowledge about autism, improve attitudes toward and perceptions of individuals with autism, and increase interest in interacting with autistic individuals (e.g., Gillespie-Lynch et al., 2015; Jones et al., 2021; Morris et al., 2020). Furthermore, such interventions have been shown to be effective across disparate groups of participants, from preschool children (Morris et al., 2020) to college students (Jones et al., 2021).

Since misinformation about autism is widespread (Aragon-Guevara et al., 2023), and accurate knowledge about autism can help non-autistic individuals be more open to positive social interactions with autistic individuals (e.g., Gillespie-Lynch et al., 2015; Jones et al., 2021; Morris et al., 2020), it is important to identify and address misinformation about autism. The current paper evaluates the extent to which college students can accurately identify the diagnostic criteria associated with autism. A better understanding of any misperceptions that exist about autistic characteristics could allow for more targeted autism knowledge interventions to be designed in the future, addressing such misperceptions.

## Metacognitive Awareness of Knowledge

Metacognition describes a person’s ability to accurately assess his/her own knowledge, including recognizing when one’s knowledge on a topic is accurate and when one’s knowledge may not be correct (Kruger & Dunning, 1999). In an influential study, Kruger and Dunning (1999) found that participants who were less knowledgeable about a topic had difficulty judging the accuracy of their knowledge and were often overconfident in their responses. Participants who were more knowledgeable about a topic had greater metacognitive awareness of their knowledge, although they tended to underestimate their knowledge. This Dunning-Kruger effect also seems to occur with autism knowledge (McMahon et al., 2020), such that participants’ perceived autism knowledge is not predictive of their actual autism knowledge. Participants who are less knowledgeable about autism are more likely to overestimate their knowledge whereas participants who are more knowledgeable about autism are more likely to underestimate their knowledge. While several studies have shown that perceived autism knowledge is not predictive of actual autism knowledge (e.g., McMahon et al., 2020; Motta et al., 2018; Shawahna et al., 2017), the literature in this area is still somewhat mixed, as other studies have shown an association between perceived and actual autism knowledge (e.g., Bono et al., 2022; Luthra & Perry, 2011; Ryan et al., 2022). This mixed literature suggests a need for more critical analysis of the relationship between perceived and actual autism knowledge, including any factors that may mediate or moderate the relationship between these constructs.

Metacognitive awareness of autism knowledge is an important construct to evaluate, as a person’s ability to accurately assess when he/she is uncertain about something can affect that person’s behavioral choices. For instance, Metcalfe and Finn (2008) asked participants to learn a series of word pairs. Some participants learned via a technique that yielded more confidence in their learning (more trials early in the learning process) and other participants learned via a technique that yielded less confidence in their learning (more trials later in the learning process). While there were no differences in recall accuracy across the groups, participants with lower confidence in their learning chose to study more word pairs again; this study indicates that confidence in learning guides behavioral choices about studying, even when controlling for accuracy. Vrugt and Oort (2008) also found that students’ metacognition influenced study behavior. Students who scored higher on a metacognitive assessment were more likely to use metacognitive learning strategies, such as self-monitoring their learning, and resource management strategies, such as using their study time well. Greater use of these two study strategies was then associated with higher exam scores.

Metacognition can also affect behavioral choices in other contexts, beyond studying. For instance, in a media literacy context, Lee and Ramazan (2021) showed that the relationship between participants’ media literacy and tendency to fact-check information from online sources was mediated by their metacognitive skills. In a health information context, Fischer et al. (2023) examined how metacognitive awareness of COVID-19 beliefs affected willingness to comply with public health recommendations related to the pandemic. Controlling for correctness of beliefs, participants with more accurate metacognitive awareness of their beliefs were more willing to vaccinate against COVID-19. In a moral judgement context, Vega et al. (2021) presented participants with a moral situation. Participants were asked to make a quick judgement about the situation and rate their certainty in their response. After making the quick judgement, participants were given an opportunity to think about the moral situation more carefully and revise their judgement. Participants who expressed less certainty in their original judgement took longer to rethink about the moral situation and also made bigger changes to their initial judgement of the situation. These studies show that individuals’ metacognitive abilities can impact their tendency to re-evaluate situations, as well as the behavioral choices and decisions made in these situations.

While research on metacognitive awareness of autism knowledge is still in a nascent stage, the literature from these other contexts suggests that metacognition can play an important role in shaping real-world behaviors. As such, individuals who are aware that their autism beliefs may not be accurate may be more likely to re-evaluate their judgement of a scenario or seek out additional information before making a decision based on these beliefs. As an example, an employer who is interviewing an autistic applicant for a job in customer service may believe that autistic individuals have social anxiety. If the employer tentatively holds this belief and recognizes that it could be an overgeneralization, that employer may seek out additional information about autism or the job applicant before making a hiring decision. An employer who confidently (but erroneously) believes that all autistic individuals are socially anxious may not re-evaluate this belief before deciding whether to hire the applicant.

## Current Study

In the current study, participants were presented with a series of characteristics that matched the diagnostic criteria for autism or another disability in the Diagnostic and Statistical Manual of Mental Disorders, Fifth Edition (DSM-5; American Psychiatric Association, 2013, 2022). Participants evaluated whether each characteristic could or could not be used to diagnose someone with autism. Next, they rated how confident they were in their response to each characteristic.

The goals of the current study were twofold. First, this study aims to assess what information college students do and don’t know about the diagnostic criteria for autism. In particular, this study evaluates how accurately students can recognize characteristics of autism as being diagnostic for autism and can recognize characteristics of other disabilities as not being diagnostic for autism. This study further assesses which disability characteristics are most difficult for students to differentiate from autism. Second, this study examines metacognitive awareness of autism knowledge, specifically the extent to which college students can accurately calibrate their confidence in their beliefs about characteristics of autism. As misinformation about autism is common (Aragon-Guevara et al., 2023), metacognitive awareness of autism knowledge may be critical for combatting false or overgeneralized perceptions of autism.

## Methods

### Participants

Participants were undergraduate students in an introductory psychology class at a midwestern university; they received course credit for participation in this study. In order to participate in the study, students had to be at least 18 years old. The current study was part of a larger research project in which students contributed data across several different timepoints. The initial sample size consisted of 426 students. Participants were excluded from this study for the following reasons: they did not participate in the study timepoint in which this data was collected (*n* = 33); they did not respond correctly to the Instructional Manipulation Check (see Measures below; *n* = 93); they did not answer and provide certainty ratings for at least 50% of the items on the Autism Symptomatology Knowledge Assessment (ASKA; *n* = 4); and they chose the same response and/or gave the same certainty rating for all items on the ASKA (*n* = 13). Therefore, the final sample size included 283 participants. See participant demographic information in Table 1.

**Table 1:** Participant demographic characteristics

Variable	*N* or M	% or SD
Sex		
Female	164	58%
Male	118	42%
Hispanic/Latino Ethnicity	11	4%
Race		
White	238	84%
Asian	37	13%
Black/African American	14	5%
American Indian/Alaska Native	3	1%
Other	3	1%
Year in School		
Freshman	151	53%
Sophomore	69	24%
Junior	36	13%
Senior/Super Senior	26	9%
Autism Experience		
Identifies as Autistic	13	5%
Knows Someone with Autism	209	74%
Major in Psychology/Psychological Science	50	18%
Age	19.28	1.51
Time Spent Interacting with Autistic People	2.18	1.16
Familiarity with Autistic Characteristics	2.59	1.04

Note: One participant did not complete any demographic information. Participants could select more than one race, so percentages for this category do not total 100%

### Measures and Procedure

This study was approved by the university Institutional Review Board. Participants signed up for the study using the SONA participant management software and completed a consent form for the study in-person. Across multiple days, participants completed a series of surveys online using the Qualtrics software. Assessments relevant to the current study are described below.

#### Autism Symptomatology Knowledge Assessment (ASKA; McMahon et al., 2020)

This assessment contains 25 characteristics that are associated with the diagnostic criteria for a disability described in the DSM-5 (American Psychiatric Association, 2013, 2022). Ten characteristics are diagnostic for Autism Spectrum Disorder: five are taken from Domain A in the DSM-5 that describes difficulties in social communication and interaction (SCI) and five are taken from Domain B in the DSM-5 that describes restricted and repetitive patterns (RRP). Fifteen characteristics are diagnostic of other disabilities in the DSM-5, with three characteristics describing each of the following disabilities: Attention-Deficit/Hyperactivity Disorder (ADHD), Generalized Anxiety Disorder (anxiety), Specific Learning Disorder (learning disability), Major Depressive Disorder (depression), and Oppositional Defiant Disorder (ODD). For each characteristic, participants were asked to identify whether that trait could be used to diagnose a person with Autism Spectrum Disorder (yes or no). Then, participants were asked to rate how certain they were in their response on a scale from “very uncertain” (1) to “very certain” (5). This assessment has shown good reliability for non-autistic characteristics (*α* = 0.80) and acceptable reliability for autistic characteristics (α = 0.78) in prior research (McMahon et al., 2020). In the current study, the reliability for other disability characteristics was acceptable (*α* = 0.70), and the reliability for autistic characteristics was marginally acceptable (*α* = 0.66).

#### Instructional Manipulation Check (Goodman et al., 2013)

In this data quality check, participants were asked to identify the topic of the current study. Participants had four response options, including a response that accurately described the study and an “other” option in which participants could write-in their own description of the study. The study directions asked participants to ignore the response option that matched the study content and instead write the words “Decision Making” into the “other” response option. Participants were excluded from the study if they did not pass this instructional manipulation check.

#### Demographics Questionnaire

Participants completed a brief questionnaire to collect demographic information, such as age, race, ethnicity, and year in college. Participants reported whether they had an autism diagnosis and whether they personally knew someone with an autism diagnosis (e.g., family member, friend, classmate, acquaintance). Participants also used a 5-point (1-5) Likert scale to indicate whether they spent a significant amount of time interacting with people with autism and whether they were familiar with the characteristics and behaviors of individuals with autism. Higher scores indexed greater perceived experience/familiarity with autism.

### Data Analysis Plan

First, the data were analyzed to determine how well students could recognize characteristics of autism as diagnostic for autism and characteristics of other disabilities as not diagnostic for autism. Accuracy scores were averaged separately for autistic characteristics and other disability characteristics, and a dependent-samples *t*-test was used to compare accuracy across these two domains. Next, to identify which disability characteristics were easiest and most challenging to categorize, accuracy scores were averaged separately for autism SCI, autism RRP, ADHD, anxiety, learning disability, depression, and ODD characteristics. A dependent samples *t*-test compared accuracy ratings across SCI and RRP autistic characteristics. A repeated-measures ANOVA compared accuracy ratings across ADHD, anxiety, learning disability, depression, and ODD characteristics.

To examine metacognitive awareness of autism knowledge, accuracy and certainty ratings were correlated for each characteristic on the ASKA. Positive correlations indicated strong metacognitive awareness, such that participants were more confident when accurately categorizing that characteristic. Negative correlations indicated weak metacognitive awareness, such that participants were more confident when *incorrectly* categorizing that characteristic.

## Results

Participants were significantly better at identifying characteristics of autism as diagnostic for autism (*M* = 0.85, *SD* = 0.17) and had more difficulty recognizing that characteristics of other disabilities were not diagnostic for autism (*M* = 0.58, *SD* = 0.21), *t*(282) = 15.61, *p* < 0.01. The three characteristics that participants most accurately categorized were: *“Difficulties adjusting behavior to suit various social contexts”*, *“Poorly integrated verbal and nonverbal communication”*, and *“Abnormalities in eye contact and body language”* (American Psychiatric Association, 2013, 2022). The three characteristics that participants least accurately categorized and often misidentified as diagnostic for autism were: *“Often has difficulty sustaining attention in tasks”*, *“Muscle tension due to excessive anxiety”*, and *“Inaccurate or slow and effortful word reading”* (American Psychiatric Association, 2013, 2022). See Table 2.

**Table 2:** Accuracy scores, the correlation between accuracy scores and certainty ratings, and certainty ratings when responding accurately and inaccurately to items on the Autism Symptomatology Knowledge Assessment

Item	DSM-5 Disability	Accuracy		Accuracy/ Certainty Correlation	Certainty when Accurate		Certainty when Inaccurate	
		M	SD		M	SD	M	SD
**Difficulties adjusting behavior to suit various social contexts**	**Autism SCI**	**0.96**	**0.19**	0.25*	**4.18**	**0.93**	**2.90**	**0.99**
**Poorly integrated verbal and nonverbal communication**	**Autism SCI**	**0.93**	**0.26**	0.32*	**3.85**	**0.95**	**2.62**	**1.12**
**Abnormalities in eye contact and body language**	**Autism SCI**	**0.91**	**0.28**	0.23*	**3.79**	**1.01**	**2.96**	**1.17**
**Failure to initiate or respond to social interactions**	**Autism SCI**	**0.91**	**0.28**	0.31*	**3.86**	**0.94**	**2.76**	**1.20**
**Hyper- or hypo-reactivity to sensory input**	**Autism RRP**	**0.88**	**0.33**	0.28*	**3.67**	**1.05**	**2.74**	**1.02**
**Highly restricted, fixated interests that are abnormal in intensity or focus**	**Autism RRP**	**0.88**	**0.33**	0.29*	**3.72**	**1.07**	**2.74**	**0.96**
**Often deliberately annoys others**	**ODD**	**0.82**	**0.39**	0.23*	**3.46**	**1.20**	**2.75**	**1.06**
**Failure of normal back-and-forth conversation**	**Autism SCI**	**0.80**	**0.40**	0.40*	**3.57**	**0.98**	**2.52**	**1.00**
**Stereotyped or repetitive motor movements**	**Autism RRP**	**0.76**	**0.43**	0.32*	**3.37**	**1.07**	**2.54**	**1.04**
Difficulties with mathematical reasoning	Learning Disability	0.76	0.43	-0.02	3.05	1.09	3.10	1.09
**Inflexible adherence to routines**	**Autism RRP**	**0.75**	**0.43**	0.34*	**3.68**	**1.10**	**2.76**	**1.10**
Depressed mood	Depression	0.74	0.44	-0.05	2.75	1.12	2.88	1.17
Feelings of worthlessness or excessive or inappropriate guilt	Depression	0.71	0.45	-0.11	2.67	1.18	2.95	1.25
**Insistence on sameness**	**Autism RRP**	**0.69**	**0.46**	0.50*	**3.81**	**1.13**	**2.45**	**0.98**
*Often argues with authority figures*	*ODD*	*0.68*	*0.47*	-0.17*	*2.70*	*1.16*	*3.14*	*1.19*
*Is often forgetful in daily activities*	*ADHD*	*0.67*	*0.47*	-0.17*	*2.77*	*1.01*	*3.14*	*1.01*
*Difficulties with spelling*	*Learning Disability*	*0.67*	*0.47*	-0.16*	*2.60*	*1.04*	*2.97*	*1.10*
Markedly diminished interest or pleasure in all, or almost all, activities	Depression	0.64	0.48	-0.03	2.91	1.03	2.98	1.08
Is often angry and resentful	ODD	0.60	0.49	< -0.01	2.90	1.04	2.90	1.15
*Sleep disturbance associated with anxiety and worry*	*Anxiety*	*0.46*	*0.50*	-0.16*	*2.71*	*1.19*	*3.08*	*1.14*
*Feeling keyed up or on edge*	*Anxiety*	*0.43*	*0.50*	-0.32*	*2.52*	*0.97*	*3.24*	*1.08*
*Is often “on the go*,*” acting as if “driven by a motor”*	*ADHD*	*0.42*	*0.49*	-0.26*	*2.64*	*1.10*	*3.24*	*1.11*
*Inaccurate or slow and effortful word reading*	*Learning Disability*	*0.42*	*0.49*	-0.22*	*2.65*	*1.10*	*3.17*	*1.12*
*Muscle tension due to excessive anxiety*	*Anxiety*	*0.40*	*0.49*	-0.28*	*2.48*	*1.07*	*3.12*	*1.09*
*Often has difficulty sustaining attention in tasks*	*ADHD*	*0.29*	*0.46*	-0.32*	*2.61*	*1.11*	*3.41*	*1.09*

Note. Bolded items show a significant positive correlation between accuracy and certainty; italicized items show a significant negative correlation between accuracy and certainty. Items on this questionnaire are taken directly or with minor modification from the Diagnostic and Statistical Manual of Mental Disorders, Fifth Edition (DSM-5; American Psychiatric Association, 2013, 2022). Autism SCI = Characteristics from domain A in the DSM-5 that describe social communication and interaction challenges in Autism Spectrum Disorder; Autism RRP = Characteristics from domain B in the DSM-5 that describe restricted and repetitive patterns in Autism Spectrum Disorder; ADHD = Attention-Deficit/Hyperactivity Disorder; Anxiety = Generalized Anxiety Disorder; Learning Disability = Specific Learning Disorder; Depression = Major Depressive Disorder; ODD = Oppositional Defiant Disorder

* *p* < 0.05

Furthermore, participants were significantly better at identifying SCI autistic characteristics (*M* = 0.90, *SD* = 0.18) compared to RRP autistic characteristics (*M* = 0.79, *SD* = 0.24), *t*(282) = 7.69, *p* < 0.01. Participants also significantly varied in the degree to which they could identify that characteristics of other disabilities were not diagnostic of autism, *F*(4, 1128) = 55.33, *p* < 0.01. Post hoc tests using the Bonferroni correction showed significant differences between all disability conditions, except for (1) ODD and depression and (2) ADHD and anxiety. Participants were most accurate in differentiating autism from ODD *(M* = 0.70, *SD* = 0.30) and depression (*M* = 0.70, *SD* = 0.33). Participants were moderately accurate in distinguishing between autism and learning disabilities (*M* = 0.61, *SD* = 0.35). Finally, participants had the most difficulty differentiating autism from ADHD (*M* = 0.46, *SD* = 0.30) and anxiety *(M* = 0.43, *SD* = 0.36).

Correlations between accuracy and certainty ratings for ASKA items showed three patterns of results: a significant positive relationship, a significant negative relationship, or no significant relationship (see Table 2). All autistic characteristics and one ODD characteristic showed a significant positive relationship between confidence and accuracy, such that participants who responded accurately were more confident in their responses. All ADHD traits, all anxiety traits, two learning disability traits, and one ODD trait showed a significant negative association between confidence and accuracy, such that participants who responded *inaccurately* were more confident in their responses. Finally, all depression characteristics, one learning disability characteristic, and one ODD characteristic did not show a significant relationship between accuracy and certainty.

## Discussion

Overall, participants had relatively high accuracy in identifying autistic characteristics as diagnostic for autism. Participants’ accuracy was particularly high for SCI autistic characteristics, wherein 4 of 5 characteristics were identified with over 90% accuracy. Participants’ high accuracy in identifying characteristics of autism is consistent with recent research in this area (Golson et al., 2022; Yu et al., 2020), suggesting that knowledge of autistic characteristics may be relatively high in the general population in the United States. Furthermore, participants’ metacognitive awareness for autistic characteristics was well-calibrated. All of the correlations between accuracy and certainty for autistic characteristics were significant and positive, indicating that participants who correctly identified these traits were more confident in their responses and participants who inaccurately categorized these traits were less certain of their responses. As less knowledgeable participants also had less confidence in their knowledge, these individuals may be more likely to re-evaluate their judgement of a situation or seek out additional information to improve their knowledge related to autistic traits.

Participants were less accurate in determining whether characteristics of other disabilities were diagnostic for autism. Participants found it easiest to differentiate autism from ODD and depression, had some difficulty distinguishing between autism and learning disabilities, and had the most difficulty differentiating autism from anxiety and ADHD. The broader research literature also suggests that college students have difficulty distinguishing between autism and other disabilities. For instance, when conducting an autism knowledge intervention, Gillespie-Lynch et al. (2015) asked participants to describe autism in their own words and found that 23% of college students confused autism with another disability. Birnschein et al. (2024) asked college students to read a vignette of a person demonstrating autistic characteristics and then provide an explanation for the vignette character’s behavior. ADHD, generalized anxiety disorder, social anxiety disorder, and learning disorder were the most frequent other disabilities that participants noted to explain the autistic characteristics in the vignette. Overall, these results indicate that college students can easily confuse autism with other disabilities, especially ADHD, anxiety, and learning disabilities. Furthermore, the current study suggests that individuals who confuse characteristics of other disabilities with autism may not be aware of their mistakes. Participants who didn’t accurately differentiate autism from ADHD, anxiety, and learning disabilities were often more confident in their knowledge than those who did accurately differentiate autism from these other disabilities. If confidence in beliefs guides behavioral choices in a real-world setting (e.g., Fischer et al., 2023; Lee & Ramazan, 2021; Metcalfe & Finn, 2008; Vega et al., 2021; Vrugt & Oort, 2008), less knowledgeable individuals, who have relatively high confidence in their beliefs, may be less likely to re-evaluate their judgement of a situation or seek out additional information to learn how autism differs from other disabilities.

Given that autism frequently co-occurs with other disabilities, including anxiety, ADHD, and learning disabilities (e.g., Lai et al., 2019; Micai et al., 2023; Valicenti-McDermott et al., 2023), it makes sense that individuals in the general population might mistake characteristics of these other disabilities for autism. Two recent systematic reviews and meta-analyses report the co-occurrence rate for autism and anxiety disorders respectively as 20% and 35% (Lai et al., 2019; Micai et al., 2023), for autism and ADHD as 28% and 37% (Lai et al., 2019; Micai et al., 2023), and for autism and learning disability as 13% (Micai et al., 2023). While these co-occurrence rates are high, the converse statistics suggest that 65-80% of autistic individuals do not have a diagnosis of anxiety, 63-72% of autistic individuals do not have ADHD, and 87% of autistic individuals do not have a learning disability. Although these estimates will likely change in future meta-analyses, these results highlight that not all individuals with autism have a co-occurring disability or have the same co-occurring disability. Furthermore, even when individuals with autism are diagnosed with a co-occurring disability, this diagnosis could change over time, as not everyone may continue to meet the diagnostic criteria for co-occurring disabilities throughout their lifetime (e.g., Lever & Geurts, 2016; McCauley et al., 2020).

If individuals in the general public perceive characteristics of other disabilities to be synonymous with autism, it may magnify the marginalization of autistic individuals. For example, unemployment and underemployment are significant problems in autism (Frank et al., 2018; Harvery et al., 2021), and these problems may be exacerbated if employers automatically presume that individuals with autism have traits associated with multiple different disabilities. Indeed, research suggests that accurate employer knowledge and understanding of autism impacts whether autistic individuals receive and/or maintain positive employment opportunities (McMahon et al., 2021; Romualdez et al., 2021; Wen et al., 2023). Additionally, misconceptions about the traits associated with an autism diagnosis may make it difficult for employers to recognize strengths in autistic job applicants. For instance, in the present study, 71% of participants thought that difficulty sustaining attention in tasks was associated with the diagnostic criteria for autism. While some individuals with autism do have difficulty sustaining attention in tasks, the ability to focus and pay attention to details are strengths for other autistic individuals (e.g., Clark & Adams, 2020; Cope & Remington, 2022; Dupuis et al., 2022; Russell et al., 2019). In their review of autistic individuals in the workplace, Bury et al. (2020) caution against applying overgeneralizations to autistic workers (e.g., all autistic individuals have impairments in attention, all individuals with autism have attentional strengths). Rather, they suggest an individual difference approach that captures the unique strengths and support needs of every autistic person. If employers automatically presume that autism is associated with other disability characteristics, employers may be less likely to take an individualized approach to determining job fit for autistic applicants/employees.

Consistent with McMahon et al. (2020), the results of the current study indicate that perceived and actual autism knowledge are separate constructs. Furthermore, these results shed light on the discrepant findings in the research literature (e.g., Bono et al., 2022; Luthra & Perry, 2011; McMahon et al., 2020; Motta et al., 2018; Ryan et al., 2022; Shawahna et al., 2017), suggesting that the relationship between perceived and actual knowledge might depend on question difficulty. Question difficulty in this study can be ascertained by the percentage of participants in the sample who responded correctly to each question (see Table 2). For easier questions, such as identifying autistic characteristics as diagnostic for autism, perceived and actual autism knowledge were more likely to be positively correlated (i.e., participants who perceived themselves to be more knowledgeable about autism did possess greater knowledge). Participants may struggle to accurately self-assess their knowledge for more difficult questions, such as identifying characteristics of other disabilities as not diagnostic for autism. For these questions, perceived and actual autism knowledge may be unrelated or even inversely correlated. Question difficulty has also been known to impact metacognitive monitoring in other knowledge domains. For instance, Hartwig and Dunlosky (2017) showed that students’ perceptions of their learning in an undergraduate statistics course were more closely calibrated with their accuracy for easy exam topics, compared to more difficult exam topics. While perceived and actual autism knowledge can both be important constructs to measure, this study confirms that these constructs are separate and not interchangeable. These constructs may be positively related, negatively related, or even unrelated, depending on the question context.

### Limitations and Future Directions

There are several limitations to the current study that should be noted. First, this study was conducted as part of a larger study in which participants read several brief vignettes featuring a character with autism. While these vignettes were narrative stories, and not explicitly designed to be educational, exposure to these vignettes may nonetheless have affected participants’ responses on the ASKA. As participants completed the ASKA after reading the vignettes, participants’ accuracy on this assessment may have been slightly elevated. Second, participants in this study were taking an introductory psychology course. Depending on when in the semester they completed the study, they may have already received some college-level education about autism and/or other disabilities in the DSM-5. As such, their knowledge of autism may not be representative of individuals in the general population, who may have never received or not recently received college-level instruction on autism and other disabilities. However, as participants did not self-report high knowledge/familiarity with autism on the Demographics Questionnaire, participants in this study also do not seem to have been unusually or exceptionally knowledgeable about autism. Third, in order to ensure content validity, the language on the ASKA is taken directly or with minor modifications from the DSM-5 (American Psychiatric Association, 2013, 2022). Some of the language on this assessment, however, may have been too technical for undergraduate students (McMahon et al., 2024). In particular, certain items on the ASKA (e.g., “stereotyped or repetitive motor movements”) used more psychology jargon than other items (e.g., “depressed mood”), which may have differentially impacted participants’ responses across ASKA items. Finally, although the DSM-5-TR (American Psychiatric Association, 2022) operationalizes the current diagnostic criteria for autism, it’s worth noting that this diagnostic criteria does change and evolve across time. Not everyone in the autism community agrees with the current criteria set forth by the DSM-5-TR, with many people in the autism community expressing concerns that language used in the DSM-5-TR is ableist (Bottema-Beutel et al., 2021), does not sufficiently represent the experience of all autistic individuals (e.g., females; Tsirgiotis et al., 2024), and doesn’t incorporate the personal lived experience of autism (Ratto et al., 2023). While the DSM-5-TR criteria was used to operationalize autism in the current study, this set of criteria is imperfect and will continue to change and evolve over time.

In the future, there is a need for more accurate public education about autism, particularly given the widespread misinformation and overgeneralizations about autism on social media (Aragon-Guevara et al., 2023). In particular, public education campaigns should provide clarity as to how autism is different from other disabilities. While many autistic individuals do have co-occurring disabilities, it’s important to clearly differentiate the characteristics of autism from other disabilities, such that individuals in the general public do not automatically presume that autistic individuals also share characteristics of multiple other disabilities. Even when autistic individuals have co-occurring disabilities, they may experience these disabilities differently than non-autistic individuals (e.g., Hunsche et al., 2022; Palmer et al., 2023).

Additionally, the general public may benefit from metacognitive training in terms of how to evaluate information about autism, mental health, and/or disability on social media. Aragon-Guevara et al. (2023) note that any “informational” videos on TikTok may be perceived to have similar levels of credibility by social media users, regardless of whether those videos contain accurate or misleading information. Furthermore, Fisher et al. (2015) found that participants who use the internet to gain knowledge on a topic tend to then overestimate their personal knowledge on that topic. As such, metacognitive training that encourages individuals to ask self-reflective questions about knowledge gained may be useful. For instance, social media users might ask themselves questions such as: “Is the person in this video knowledgeable about autism overall, or is this person primarily sharing a story about their personal experience with autism?”; “Is this information true for all autistic people, or only true for some people with autism?”; and “Is there any information in this video that could be misleading, overly simplistic, or exaggerated?”. Future research should examine whether such training increases metacognitive awareness, such that participants’ confidence is more closely calibrated with their actual knowledge of autism. Future public education efforts may be most effective if they have a dual focus on (1) sharing accurate information about autism and correcting misperceptions and (2) teaching metacognitive skills for individuals to self-reflect on the accuracy of “autism knowledge” presented via social media.

Finally, this research suggests that metacognitive awareness of autism knowledge may depend on the specific knowledge construct. Individuals in the general population may be able to accurately assess their knowledge for easier questions, such as recognizing the characteristics of autism. However, they may have more difficulty assessing their knowledge for challenging questions, such as differentiating characteristics of autism from those of other disabilities. Also, in future research, it’s important to examine how metacognitive awareness may impact behavioral choices. If metacognitive skills influence one’s tendency to re-evaluate a situation and/or seek additional information about autism, metacognition may be key for minimizing the impact of misinformation or overgeneralizations about autism.

In conclusion, this study shows that college students can identify autistic characteristics, especially social communication and interaction characteristics, as being diagnostic for autism with fairly high accuracy. However, college students have difficulty identifying whether characteristics of other disabilities, especially learning disabilities, ADHD, and anxiety, are diagnostic for autism. In addition, students’ accuracy on autism knowledge questions and certainty in their responses were positively correlated when evaluating autistic characteristics, but typically unrelated or inversely correlated when evaluating characteristics of other disabilities. These results confirm that perceived and actual autism knowledge are separate constructs, and their relationship may depend on question difficulty. Furthermore, these results suggest that students are not self-aware of their difficulty differentiating characteristics of other disabilities from autism, such that they may not seek out additional corrective information about autism.

## Footnotes

1 Research suggests that some individuals in the autism community prefer person-first language and other individuals in the autism community prefer identity-first language (e.g., Buijsman et al., 2023; Kenny et al., 2016). To be respectful of these different preferences, the current paper uses both person-first and identity-first language.
